# Subwavelength imaging using orbital angular momentum waves generated by metasurfaces

**DOI:** 10.1038/s41598-025-04813-8

**Published:** 2025-07-01

**Authors:** Mohammadreza Ashrafian, Leila Yousefi

**Affiliations:** 1 School of Electrical and Computer Engineering, University of Tehran, Tehran, 1417614411 Iran; 2 School of Engineering and Informatics, University of Sussex, Falmer, BN1 9RH UK

**Keywords:** Subwavelength Imaging, Diffraction Limit, Dielectric Metasurface, Laguerre-Gaussian, Orbital Angular Momentum, Spatial Frequency, Electrical and electronic engineering, Optics and photonics

## Abstract

Using conventional imaging techniques, the maximum obtainable resolution of an object is limited to about half the wavelength of the incident light, a phenomenon known as the diffraction limit. This limitation poses significant challenges in fields requiring high-resolution imaging, such as microscopy and nanotechnology. In this paper, we address this challenge by designing a dielectric metasurface to generate an optical Laguerre-Gaussian (LG) orbital angular momentum (OAM) beam. This innovative approach leverages the unique properties of OAM beams to achieve imaging beyond the diffraction limit. The dielectric metasurface is meticulously engineered to control both the phase and amplitude of the output wave, enabling the generation of high-quality LG OAM beams. Unlike traditional near-field and far-field imaging techniques, our proposed method does not necessitate a restricted distance between the imaging system components, offering greater flexibility and practicality in various applications. To validate the performance of our method, we conducted comprehensive numerical simulations. The results demonstrate that the generated OAM beam can achieve a resolution of 0.29 times the incident wavelength, surpassing the conventional diffraction limit.

## Introduction

Achieving high-resolution imaging is crucial across various scientific and research fields. However, conventional imaging techniques are constrained by the diffraction limit, which restricts resolution to approximately half the wavelength of the incident light^1,2^. This limitation arises because high spatial frequency information of an object is carried by evanescent waves, which decay exponentially. Consequently, subwavelength details of the object are inaccessible in the far-field zone^3–5^.

To surpass the diffraction limit, various methods have been proposed, categorized into near-field and far-field techniques. Near-field methods aim to capture evanescent waves, which carry subwavelength information, before their amplitude diminishes significantly. Techniques such as Near-field Scanning Optical Microscopes (NSOM)^6,7^ rely on placing detectors very close to the object to record evanescent waves, while superlens structures amplify evanescent waves using materials like silver, which has negative permittivity at optical wavelengths^8,9^. However, these methods are limited by the complexity of getting close to the object and the time-consuming nature of scanning the imaging area.

Far-field methods aim to convert evanescent waves into propagating waves, making subwavelength information accessible at a distance. One early far-field method is the far-field superlens, introduced in^10^. These periodically corrugated superlenses can convert evanescent waves into propagating ones, eliminating the need to place detectors near the superlens. Another far-field approach is the hyperlens, proposed in^11^, which uses hyperbolic metamaterials. These materials have a hyperbolic dispersion equation under certain electromagnetic modes, enabling the conversion of evanescent waves to propagating waves. Metasurfaces offer another far-field solution. By designing unit cells that apply engineered phase shifts to incident waves, metasurfaces can convert evanescent waves into propagating waves, transferring high spatial frequency information to the far zone^12,13^.

A significant limitation of all far-field subwavelength imaging methods is that, although there is no need to place detectors very close to the object, the structure which converts evanescent waves to propagating ones must be in the near field of the object to perform the conversion before the amplitude of evanescent waves decreases sharply^10–15^.

In this study, we propose a new method to overcome the diffraction limit and create far-field high-resolution images using orbital angular momentum (OAM) waves as the incident light source. By employing OAM waves, there is no need to design a structure for the conversion operation, thus eliminating the limitation of placing that structure very close to the object.

In previously reported works, OAM beams have been used to improve edge contrast in images^16–18^ and to enhance image resolution through localized regions, or “hot spots,” generated by the superoscillation phenomenon^19,20^. However, edge-contrast imaging techniques using OAM beams are limited to highlighting object edges^16–18^, as their spatial structure makes them insensitive to uniform regions, which prevents full object reconstruction. In superoscillation imaging, a beam such as a radially polarized Laguerre-Gaussian beam is focused to create a hot spot smaller than the diffraction limit, typically surrounded by side lobes^19,20^. The resulting point spread function (PSF) is then used to numerically reconstruct the image of an object. However, the side lobes of the focused beam may cause unwanted effects when imaging general objects. Additionally, due to the subwavelength size of the hot spots, the imaging process can be time-consuming. Unlike superoscillation imaging, our proposed method does not focus the LG beam. Instead, the key distinction of our approach lies in utilizing the shifting property that the LG beam provides in the Fourier spectral domain. In^21^, it is shown that by using about twenty OAM modes, images with subwavelength resolution are achievable. However, the method presented in^21^ is not practical as numerous modes are required to create the image. Moreover, high-order Bessel beams have been used for imaging^22^, but the resolution does not exceed the diffraction limit, as the proposed method produces point-like spread functions that are larger than the diffraction limit. For practical applications, accurate detection of OAM beams is crucial. Metasurface-based detection schemes have demonstrated compact and efficient recognition of OAM states^23–26^, supporting the development of integrated imaging and communication platforms. Additionally, coherence-based detection methods offer a complementary approach to robust OAM detection under challenging propagation conditions^27^.

Here, we propose a method in which a single Laguerre-Gaussian (LG) OAM beam is used to break the diffraction limit and increase the resolution beyond it. In the proposed method, the object and image planes are both located in the far-field zone of the source, and unlike previously reported works that require a limited distance between imaging system parts, this limitation does not exist in the proposed technique. In the proposed method, a plane wave is illuminated on a dielectric metasurface engineered to create Laguerre-Gaussian (LG) OAM beams when excited by a plane wave. The generated Laguerre-Gaussian (LG) OAM beams then shine on the object. The scattered wave from the object, after passing through a standard optical lens, creates the image. The generated image needs to undergo post-processing analysis to result in the final image. This post-processing is required because OAM waves shift the Fourier transform of the object in the k-space, and this shift needs to be compensated through post-processing analysis.

The structure of the paper is as follows: First, we explain the proposed method through mathematical equations and numerically prove that using the proposed approach can break the diffraction limit and increase the resolution beyond it. Then, the structure of the metasurface used to generate the OAM beam and the design method is explained. The performance of the designed metasurface in generating Laguerre-Gaussian (LG) OAM beams is then verified through numerical full-wave simulation. Finally, the generated beam of the metasurface is used for superresolution imaging, and the results are presented and discussed.

## Background theory and the proposed method

**Fig. 1:**
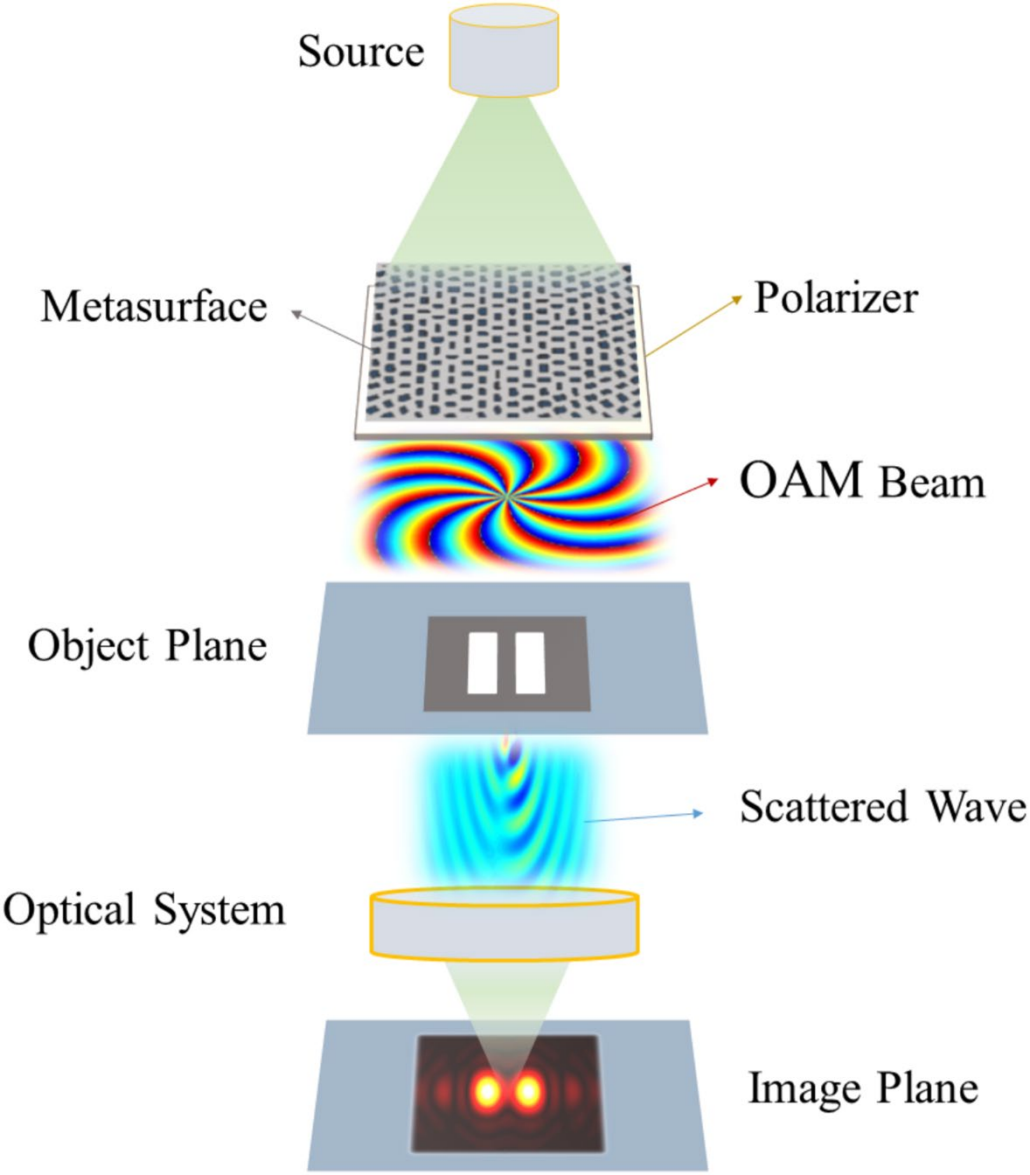
A schematic diagram of the proposed imaging setup where an OAM beam generated by a dielectric metasurface with a polarizer in its near field can transfer the subwavelength information to the far zone. Specific areas of the beam with considerable intensity as the incident waves cause the high spatial frequency information of the object be carried by propagating scattered waves instead of the evanescent ones leading to the resolution improvement.

Figure 1 shows the conceptual schematic diagram of the proposed imaging setup. As shown in this figure, in our proposed method, a Laguerre-Gaussian (LG) OAM beam produced by a dielectric metasurface is used for creating the image. In the following parts, we prove that using the OAM wave can transfer the subwavelength information of the object to the far zone through providing shift in the spectral Fourier domain resulting in an image with the resolution beyond the diffraction limit. Using conventional imaging methods with a uniform plane beam or Gaussian beam as the incident wave, subwavelength information of the object is not receivable in the far zone and the resolution would be limited to the diffraction limit.

When shining an object with an incident wave to make an image, part of the wave will be scattered from the object. Using the first-order Born approximation, the scattered field can be written as^21^:

1 $$\begin{aligned} E_{sca}(\vec {r}) = \iiint _{-\infty }^{+\infty } G(\vec {r}-\vec {r}\hspace{1.111pt}\phantom {r}') \, O_{3D}(\vec {r}\hspace{1.111pt}\phantom {r}') \, E_{in}(\vec {r}\hspace{1.111pt}\phantom {r}') \, \text {d}\vec {r}\hspace{1.111pt}\phantom {r}' \end{aligned}$$

where $G(\vec {r}-\vec {r}\hspace{1.111pt}\phantom {r}') = \frac{e^{i k_{0} |\vec {r} - \vec {r}\hspace{1.111pt}\phantom {r}'|}}{4 \pi |\vec {r} - \vec {r}\hspace{1.111pt}\phantom {r}'|}$ is the free space Green’s function with wavenumber $k_0 = 2 \pi / \lambda _0$ and with $\lambda _0$ being the wavelength of the incident wave, and $O_{3D}(\vec {r})$ is the object function defining the properties of the object in the medium.The object function can be written as $O_{3D}(x,y,z)=O(x,y)\, \delta (z)$, as we are dealing with two-dimensional (2D) imaging systems. By this assumption and using (1), the spatial Fourier transform (spectrum) of the scattered waves $\tilde{E}_{sca}(k_{x},k_{y})$, can be calculated as:

2 $$\begin{aligned} \tilde{E}_{sca}(k_{x},k_{y}) = \frac{i}{2} \frac{e^{ik_{z} |z_r|}}{k_{z}} \, \iint _{-\infty }^{+\infty } \, E_{in}(x',y') \, \times O(x',y') \; e^{ -ik_{x} x'-ik_{y} y'} \; \text {d}x' \text {d}y' \end{aligned}$$

which is rewritten as:

3 $$\begin{aligned} \tilde{E}_{sca}(k_{x},k_{y}) = \frac{i}{2} \frac{e^{ik_{z} |z_r|}}{k_{z}} \, \tilde{E}_{in}(k_{x},k_{y}) \, \otimes \, \tilde{O}(k_{x},k_{y}) \end{aligned}$$

where $z_r$ is the longitudinal distance from the object, $\tilde{E}_{in}(k_{x},k_{y})$ and $\tilde{O}(k_{x},k_{y})$ are the Fourier transform (spectrum) of the incident wave and object, respectively, and $\otimes$ denotes the convolution operator.

As shown in (3), since the object spectrum is convoluted with the spectrum of the incident field to create the image spectrum, by suitable engineering of the incident field spectrum, one can shift the information involved in the evanescent part of the spectrum ($k_x^2+k_y^2>k_0^2$) to the propagating part of it ($k_x^2+k_y^2<k_0^2$). For the incident wave, we intend to employ Laguerre-Gaussian (LG) beams which are expressed by the following equation^28^:

4 $$\begin{aligned} LG_{p,\ell }(\rho ,\phi ,z)=\frac{w_0}{w(z)} \text {exp}(\frac{-\rho ^{2}}{w^{2}(z)}) \text {exp}(\frac{ikz \rho ^{2}}{2(z^{2}+z_{R}^{2})}) (\frac{\rho \sqrt{2}}{w(z)})^{|\ell |} L_{p}^{|\ell |}(\frac{2 \rho ^{2}}{w^{2}(z)}) \, \, \text {exp}(-i(2p+\ell +1) \tan ^{-1} \frac{z}{z_{R}}) \, \, \text {exp}(i \ell \phi ) \end{aligned}$$

where $w(z) = w_{0} \sqrt{(z^{2}+z_{R}^{2})/(z_{R}^{2})}$, $w_0$ is waist radius, $z_R$ is Rayleigh range, $L_{p}^{|\ell |}(\cdot )$ denotes associated Laguerre polynomial, and the radial index *p* is the number of radial nodes in the amplitude distribution. Also, the azimuthal index $\ell$ is called topological charge which defines OAM mode order.

**Fig. 2:**
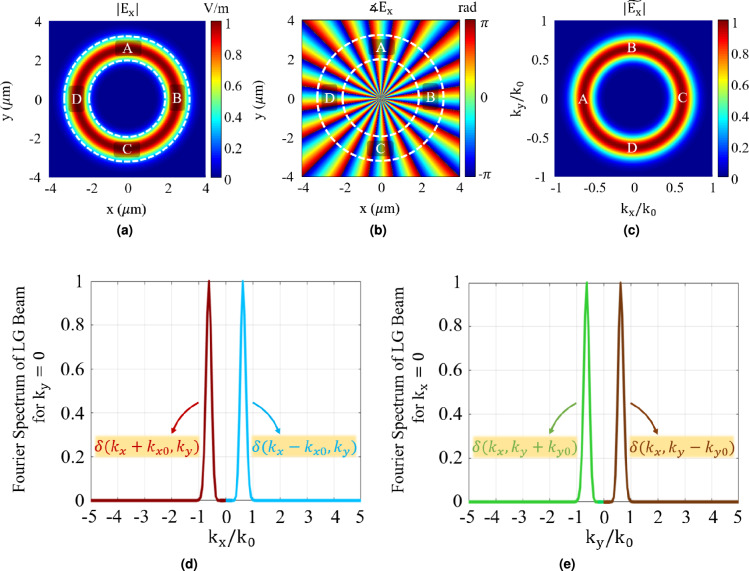
(**a**) Amplitude, (**b**) Phase, and (**c**) Normalized spatial spectrum of a Laguerre-Gaussian (LG) OAM beam with mode order of $\ell = 20$, radial index of $p = 0$ and waist radius of $w_0 = 0.8\ \mu m$. (**d**) Normalized one dimensional LG Beam’s spatial spectrum along $k_x$ when $k_y = 0$ which depicts how A and C areas locally model $\delta (k_x+k_{x0},k_y)$ and $\delta (k_x-k_{x0},k_y)$, respectively, with $k_{x0} \approx 0.6 k_0$. (**e**) Normalized one dimensional LG Beam’s spatial spectrum along $k_y$ when $k_x = 0$ which depicts how B and D areas locally model $\delta (k_x,k_y-k_{y0})$ and $\delta (k_x,k_y+k_{y0})$, respectively, with $k_{y0} \approx 0.6 k_0$.

Figure 2 depicts a LG beam with mode order $\ell = 20$, radial index $p = 0$ and waist radius $w_0 = 0.8\ \mu m$, at the wavelength of 500 nm. Figure 2cshows the derived spatial spectrum of this LG beam, where points A, B, C, and D marked in the figure correspond to the areas labeled A, B, C, and D in Figs. 2aand 2b. Furthermore, the one dimensional spatial spectrum of the LG beam along $k_x$ and $k_y$ axis is depicted in Figs. 2dand 2e, respectively. As shown in these figures, A area locally models $\delta (k_x+k_{x0},k_y)$ in the Fourier spectral domain, while C area locally models $\delta (k_x-k_{x0},k_y)$, with $k_{x0} \approx 0.6 k_0$. Additionally, B area locally models $\delta (k_x,k_y-k_{y0})$, while D area locally models $\delta (k_x,k_y+k_{y0})$, with $k_{y0} \approx 0.6 k_0$. Therefore, using A and C areas of the LG beam as the incident waves can transfer the subwavelength information of the object, carried by $(k_0, 1.6 \ k_0)$ and $(-1.6 \ k_0, -k_0)$ evanescent components, respectively, to the propagating region in the Fourier spectral domain. Similar phenomenon is observable in the $k_y$ domain.

**Fig. 3:**
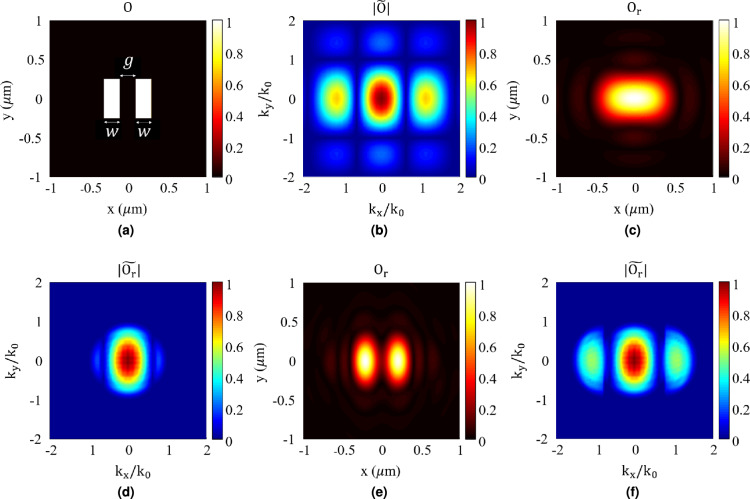
(**a**) Object with two slits, each having a vertical length of $\lambda$, and both slit width (*w*) and gap (*g*) equal to $0.4\lambda$ and (**b**) Normalized spatial spectrum of the object. (**c**) Recovered image and (**d**) Normalized recovered spatial spectrum of the object using only plane wave. (**e**) Recovered image and (**f**) Normalized recovered spatial spectrum of the object using A and C areas of the LG beam depicted in Fig. 2b.

In order to demonstrate the capability of the proposed technique, here, we use it to make an image of the object shown in Fig. 3a. This object consists of two subwavelength slits with a gap in between. The object involves subwavelength features leading to creation of evanescent waves with lateral wave numbers higher than $k_0$ as clearly shown in Fig. 3bwhich illustrates the Fourier transform of the object in the spectral domain. Therefore, when using conventional imaging techniques, these feature sizes can’t be recovered accurately. To illustrate this fact, a FDTD simulation is carried out in Ansys Lumerical. In this simulation, the object is illuminated by a uniform plane wave with the wavelength of 500 nm, and the scattered field from the object is recorded at the far zone region at the distance of $4\lambda$ from the object. Then the Fourier spectral transform of the image is recovered using the following equation which has been derived from the Equation (3) with the assumption of $\tilde{E}_{in}(k_{x},k_{y})$ equal to $\delta (k_x,k_y)$, as the incident wave is a pure plane wave:

5 $$\begin{aligned} \tilde{O}(k_{x},k_{y}) = \frac{2}{i} \frac{k_{z}}{e^{ik_{z} |z_r|}} \, \tilde{E}_{sca}(k_{x},k_{y}) \end{aligned}$$

The result of this calculation is shown in Fig. 3d. Comparing the recovered spectrum in this figure with the actual spectrum of the object in Fig. 3bclearly demonstrates that only the part of the spectrum within the region of propagating waves ($k_x^2+k_y^2<k_0^2$) has been recovered, while other parts of the spectrum, specifically the evanescent waves ($k_x^2+k_y^2>k_0^2$), have been omitted. The effect of this spatial filtering is evident in Fig. 3c, which illustrates the recovered image achieved by applying the inverse Fourier transform to the results of Fig. 3d. As shown, the two subwavelength slits are not distinguishable in the recovered image, clearly demonstrating the diffraction limit phenomenon.

For comparison and to show the effect of using OAM waves for imaging, we repeat the explained imaging process using the A and C parts of Laguerre-Gaussian (LG) OAM beam depicted in Fig. 2 as the incident waves. The results are shown in Figs. 3f, 3e. Fig. 3eillustrates the resultant image, while Fig. 3fdepicts the spatial Fourier transform of the image. As shown in Fig. 3e, the two subwavelength slits are distinguishable, proving the ability to image with a resolution higher than the diffraction limit when using the proposed technique. This is predictable from the results of Fig. 3f, which illustrates the Fourier transform of the retrieved image. As shown, the retrieved information includes evanescent waves with wave numbers higher than ($k_0$), which contain the subwavelength features of the object. Therefore, as clearly demonstrated by these results, using Laguerre-Gaussian (LG) OAM beams for imaging can overcome the diffraction limit and result in higher resolution by shifting the evanescent waves in the spectral domain and converting them into propagating ones.

## Laguerre-Gaussian OAM beam generation

**Fig. 4:**
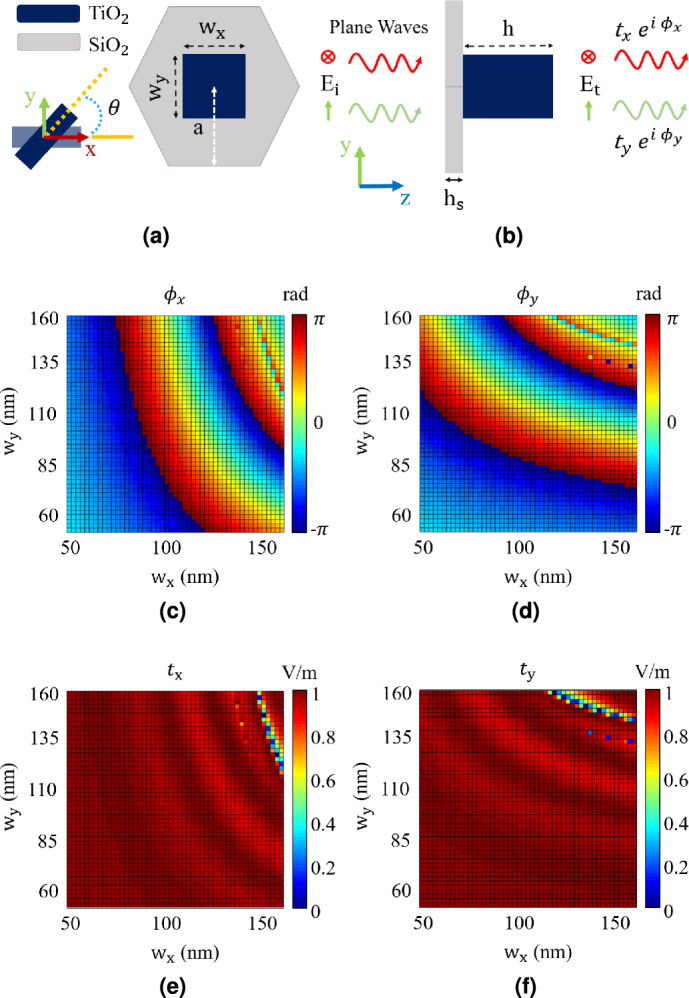
(**a**) Top view and (**b**) Side view of the proposed hexagonal unit cell made of $\text {TiO}_\text {2}$ pillar and $\text {SiO}_\text {2}$ substrate with refractive index of 2.71 and 1.46, respectively, at the wavelength of 500 nm. The periodicity or lattice constant (2a) is assumed to be 270 nm and the heights of the pillars (h) and the substrate ($\text {h}_\text {s}$) are 1000 nm and 200 nm, respectively. Transmittance phase parameters $\phi _x$, $\phi _y$, and transmittance amplitude parameters i.e. ${t}_{x}$ and ${t}_{y}$ are obtained through unit cell analysis in CST Studio software, shown in Figs. 4c-4f.

In this section, our goal is to design a dielectric metasurface capable of producing a Laguerre-Gaussian (LG) OAM beam to be employed in subwavelength imaging. Metasurfaces are structures with small building blocks known as unit cells, designed to manipulate and engineer optical beams by controlling the amplitude and phase of the scattered light^29–35^. In this work, we have designed a metasurface that is able to generate a Laguerre-Gaussian (LG) OAM beam when illuminated by a plane wave at the wavelength of 500 nm. The unit cell of the designed metasurface is shown in Fig. 4. As illustrated in this figure, the unit cell includes titanium dioxide ($\text {TiO}_{2}$) rectangular pillars with 1000 nm thickness located on a silica ($\text {SiO}_{2}$) substrate with 200 nm thickness. The lattice is hexagonal with the periodicity (lattice constant) of $2a=270\ nm$. The lattice constant has been chosen to be small enough in order to generate continuous amplitude and phase required to generate a high-order Laguerre-Gaussian (LG) OAM beam with mode order of $\ell = 20$, radial index of $p = 0$ and waist radius of $w_0 = 0.8\ \mu m$. The amplitude and phase of the transmitted beam required to generate the Laguerre-Gaussian (LG) OAM beam, is controlled by designing the lateral dimensions, $\text {w}_\text {x}$, $\text {w}_\text {y}$, and rotation of the nano-pillar of unit cell (see Fig. 4a).

As the first step of metasurface design, unit cell characterization is performed through numerical full wave electromagnetic simulation. In this simulation, the unit cells with different dimensions are illuminated by a plane wave and the transmitted wave is analyzed (see Fig. 4b). The simulation is performed using CST Studio software, and the results are shown in Figs. 4c-4f. These figures illustrate the amplitude (${t}_{x}$, ${t}_{y}$) and phase ($\phi _\text {x}$, $\phi _\text {y}$) of the transmitted wave when the unit cell is illuminated by a plane wave with the E-field in the *x* and *y* directions, respectively.

As the second step of the design, Jone’s matrix is considered which expresses the relation between the Electric field of the incident wave, $E_{in}$ and the Electric field of the transmitted wave, $E_{out}$ as follows:

6 $$\begin{aligned} E_{out}= J E_{in}, \ J = R(- \theta ) \begin{bmatrix} t_x e^{i \phi _x} & 0 \\ 0 & t_y e^{i \phi _y} \end{bmatrix} R(\theta ) \end{aligned}$$

where $R(\theta )$ is the rotation matrix with $\theta$ showing the rotation angle of the pillars. Assuming $\phi _x = \psi$ and $\phi _y = \psi + \Delta \phi$, and the incident wave has an electric field oriented in the *y* direction, Equation (6) can be rewritten as follows^31^:

7 $$\begin{aligned} \begin{aligned} \begin{bmatrix} T_x e^{i \psi _{xd}}\\ T_y e^{i \psi _{yd}} \end{bmatrix} = \begin{bmatrix} \cos {\theta } & -\sin {\theta } \\ \sin {\theta } & \cos {\theta } \end{bmatrix} e^{i \psi } \begin{bmatrix} 1 & 0 \\ 0 & e^{i \Delta \phi } \end{bmatrix} \begin{bmatrix} \cos {\theta } & \sin {\theta } \\ -\sin {\theta } & \cos {\theta } \end{bmatrix} \begin{bmatrix} 0 \\ 1 \end{bmatrix}\\ \end{aligned} \end{aligned}$$

where $\text {exp}(i \psi _{xd})$ and $\text {exp}(i \psi _{yd})$, and the coefficients $T_x$ and $T_y$ are determined based on the phase and amplitude profiles of the output LG beam, respectively. Note that in the above equation it is assumed that $t_x \approx t_y \approx 1$, and $T_x^2+T_y^2$ is equal to one based on the conservation of energy. Equation (7) forms a non-linear system of four equations with three unknowns, which may not have any solution. To be able to find solution to this equation, we assume that $\Delta \phi$ is equal to $\pi$, which means that we select pillars that generate waves with $\pi$ phase difference when excited by waves with *x*-directed and *y*-directed E-fields (see Fig. 4). Furthermore, as we are using a polarizer after the metasurface (see Fig. 1) which eliminates *y* component of the resultant field, $\psi _{yd}$ would not be important in Equation (7), however, it is assumed to be equal to $\psi _{xd}$. Using these assumptions and after some mathematical simplifications, Equation (7) results in $\sin {2\theta } = T_x$ and $\psi = \psi _{xd}$. Therefore, the amplitude of *x* component of the LG beam determines the rotation of the pillar of each unit cell, while the phase profile of the desired LG beam, $\psi _{xd}$ along with the assumption $\Delta \phi = \pi$, determines the dimensions of the pillars (see Fig. 4).

**Fig. 5:**
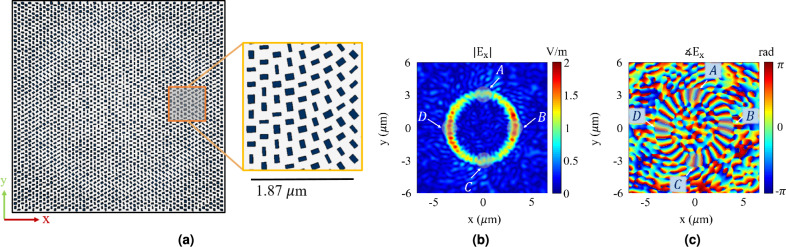
(**a**) Synthesized metasurface, to produce the desired LG beam with mode order of $\ell = 20$, radial index of $p = 0$ and waist radius of $w_0 = 0.8\ \mu m$. (**b**) Amplitude and (**c**) Phase of the generated *x* component field at the distance of $10\lambda$ from the metasurface.

**Fig. 6:**
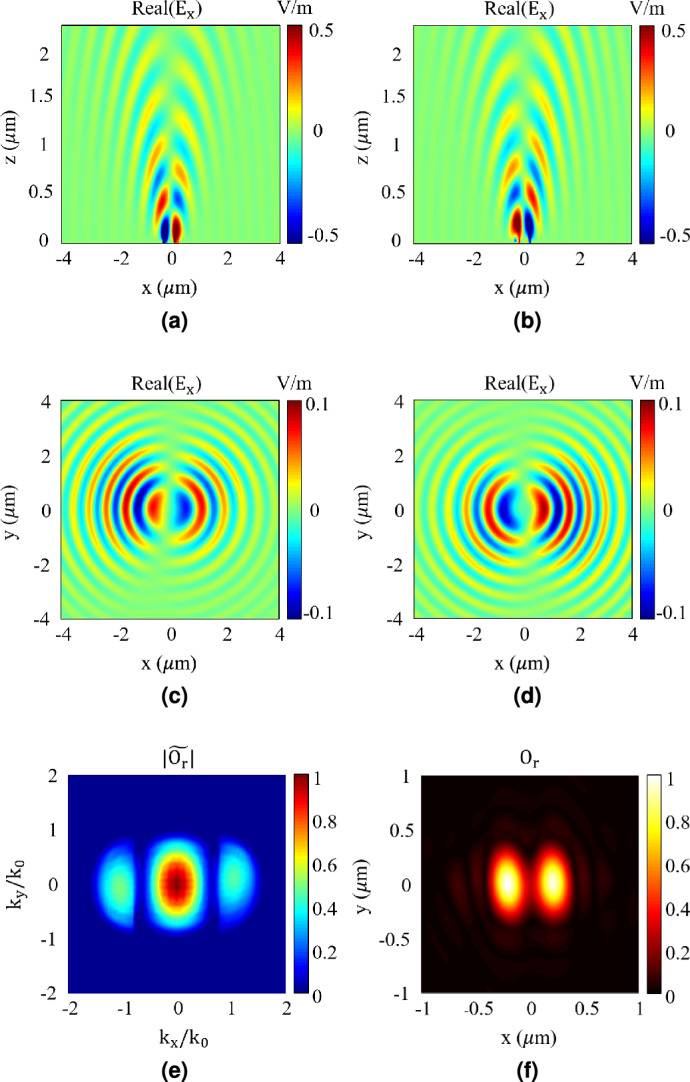
(**a**) and (**b**) Scattered fields from the object along propagation axis on $y=0$ plane, using *A* and *C* areas depicted in the amplitude profile in Fig. 5b as the incident waves, respectively, (**c**) and (**d**) Recorded scattered fields on $x-y$ plane at the distance of $4\lambda$ from the object using *A* and *C* areas depicted in the amplitude profile in Fig. 5b as the incident waves, respectively, (**e**) Recovered normalized spatial spectrum of the object with $w = g = 0.4\lambda$ and (**f**) Recovered image of the object.

**Fig. 7:**
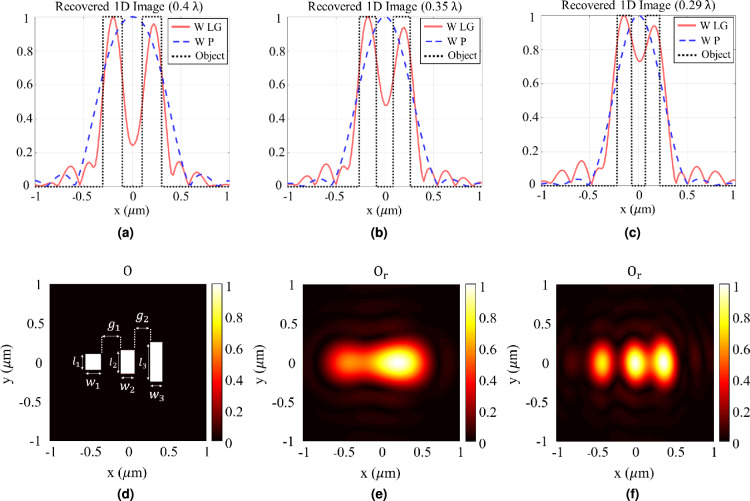
(**a**–**c**) Normalized recovered 1D images of objects with (**a**) $w = g = 0.4\lambda$, (**b**) $w = g = 0.35\lambda$, and (**c**) $w = g = 0.29\lambda$. In these figures, “W”, “LG”, and “P” denote “with”, “Laguerre-Gaussian”, and “plane wave”, respectively. “W LG” indicates that our proposed method was applied for imaging, while “W P” corresponds to imaging using a plane wave. “Object” represents the ideal 1D object profile. (**d**) An object consisting of three slits with dimensions: $l_1 = 0.4\lambda$, $w_1 = 0.4\lambda$; $l_2 = 0.6\lambda$, $w_2 = 0.35\lambda$; $l_3 = \lambda$, $w_3 = 0.3\lambda$; and gaps $g_1 = 0.5\lambda$, $g_2 = 0.4\lambda$. (**e**) Normalized recovered image of the object shown in Fig. 7 d using a plane wave. (**f**) Normalized recovered image of the object shown in Fig. 7dusing our proposed method.

Using the explained analytical method and the data presented in Fig. 4, we designed a metasurface to generate a LG beam with the mode order $\ell = 20$, the radial index of $p = 0$, and waist radius of $w_0 = 0.8\ \mu m$. The resultant metasurface is shown in Fig. 5a. To verify its performance, we conducted a full-wave numerical simulation using CST Studio software. In this simulation, the metasurface was illuminated by a plane wave with the electric field in the *y* direction, and the generated field was recorded at a distance of $z = 10 \lambda$ from the metasurface. The results are shown in Fig. 5. Figure 5billustrates the amplitude of the generated beam, while Fig. 5cshows its phase. Comparing these results with the ideal LG beam shown in Fig. 2 confirms that the generated beam has an acceptable and suitable amplitude and phase profile required for imaging. In designing our proposed metasurface, we have thoroughly considered all fabrication constraints and limitations, ensuring that it can be produced using current technology. Given the similarity in material and structure, the fabrication technology used to create the metalens in^36^ is applicable to our metasurface as well.

Moreover, it is noteworthy that the dimensions of the pillars in our metasurface result in an aspect ratio of 20, which is relatively high. To guarantee the feasibility of fabricating this metasurface and using it for practical imaging, we recommend the fabrication technology presented in^36^, as it supports aspect ratios up to 37.5.

To verify the performance of the designed metasurface for imaging, the next step involves using the generated LG beam to obtain images of objects with subwavelength feature sizes. The objects under test, shown in Fig. 3a, consist of two slits with a subwavelength gap between them. The results are presented in Figs. 6 and 7. Figure 6 illustrates the results for an object with $w = g = 0.4 \lambda$. As clearly shown in this figure, although the object features are smaller than the diffraction limit, the image has been successfully recognized, proving the ability of the proposed method to overcome the diffraction limit and go beyond it. Furthermore, the results shown in Fig. 6fare very similar to those in Fig. 3e, where an ideal LG beam was used for imaging. This similarity demonstrates the acceptable accuracy of the LG beam generated by the metasurface for imaging applications. Figure 7 shows the reconstructed images of objects with different dimensions. This figure compares the image achieved using the proposed method with the real object and the reconstructed image when a plane wave is used for imaging. As shown, using a plane wave, none of the objects have been successfully reconstructed. In contrast, the proposed technique successfully recovers objects with feature sizes as small as $0.29\ \lambda$ based on the Rayleigh criterion. To further illustrate the versatility of the super-resolution imaging capability, we applied the proposed method to a more complex object featuring three slits with subwavelength dimensions, as depicted in Fig. 7d. The normalized recovered images obtained using a plane wave and our method are shown in Figs. 7eand 7f, respectively. These results demonstrate that the proposed approach can effectively resolve multiple subwavelength features.

In our proposed method, one way to further improve resolution is by increasing the topological charge $\ell$. When the beam waist $w_0$ and radial index *p* are kept constant for an LG beam, a higher topological charge introduces greater phase variation across the beam, resulting in higher spatial frequency components in the spectral domain. These higher frequencies allow for larger spectral shifts, leading to higher resolution in the resultant image. However, as the topological charge increases, it becomes more challenging to design and fabricate a metasurface to generate that OAM wave. The dimensions of the required metasurface increase, and due to higher phase variation, a smaller unit cell (lattice constant) is needed to maintain the quality of the generated beam. Therefore, in this work, we have selected the topological charge $\ell = 20$, which is large enough to achieve high resolution while still being feasible to generate using metasurfaces.

In future work, polarization control can be integrated within the same metasurface structure, eliminating the need for a separate polarizer. Drawing inspiration from recent advancements in multifunctional metasurfaces capable of simultaneous polarization manipulation and wavefront shaping^37,38^, it is feasible to design a single metasurface structure that combines OAM generation with polarization control.

## Conclusion

In this article, a new approach for subwavelength imaging was proposed and demonstrated using Laguerre-Gaussian OAM beams. It was numerically proven that OAM beams can transfer high-frequency information of the object to the far zone. To generate the Laguerre-Gaussian OAM beams, an optical metasurface was designed, and its performance was verified through full-wave numerical simulations. In the proposed approach, controlling the amplitude of the OAM beam is crucial. To achieve this, we designed a dielectric metasurface to control both the phase and amplitude of the output wave. The generated OAM beam was used for imaging, and the results showed that the proposed technique can overcome the diffraction limit, achieving a resolution of $0.29\ \lambda$ at an operating wavelength of 500 nm.

## Methods

The imaging process is carried out using Ansys Lumerical software. FDTD simulation is performed at an operating wavelength of 500 nm, with a plane wave or a user-defined field (e.g., the specified regions of the Laguerre-Gaussian OAM beam in this paper) as the incident source on the object, and the scattered fields are obtained. The surrounding boundary conditions are set as Perfectly Matched Layer (PML), and the objects, consisting of two and three slits, are modeled as vacuum slits inside a Perfect Electric Conductor (PEC) medium with a thickness of 0.1 $\lambda$.

Image reconstruction is performed in MATLAB, where the codes are written based on the mathematical equations presented in the paper.

The design of the dielectric metasurface and the extraction of its generated field are carried out using CST Studio software. First, unit cell analysis is conducted using the Frequency Domain Solver with all-mode Floquet excitation as the source type. The boundary conditions are set as “unit cell” along the *x*- and *y*-axes and “open (added space)” along the *z*-axis. By sweeping the unit cell’s nano-pillar dimensions ($\text {w}_\text {x}$, $\text {w}_\text {y}$) from 50 to 160 nm at a wavelength of 500 nm, transmittance phases and amplitudes are extracted. Then, following the procedure described in the paper, the dimension and rotation of each pillar throughout the metasurface are specified and stored in MATLAB. Using MATLAB and CST Studio interconnection, the metasurface is designed. In the final step, full-wave simulation is conducted using the Time Domain Solver for the designed metasurface at 500 nm, and the resultant fields are extracted. Additionally, the *x*-directed field is used as the incident source in Ansys Lumerical for the imaging process.

## Data Availability

The dataset used and/or analyzed during the current study are available from the corresponding author on reasonable request.

## References

[1] Abbe, E. Beitrge zur theorie des mikroskops und der mikroskopischen wahrnehmung. *Archiv fr Mikroskopische Anatomie* **9**, 413–418. 10.1007/BF02956173 (1873).

[2] Born, M. et al. *Principles of Optics: Electromagnetic Theory of Propagation, Interference and Diffraction of Light* (Cambridge University Press, 1999), 7 edn.

[3] Zhang, X. & Liu, Z. Superlenses to overcome the diffraction limit. *Nature materials* **7**, 435–41. 10.1038/nmat2141 (2008). 18497850 10.1038/nmat2141

[4] Lu, D. & Liu, Z. Hyperlenses and metalenses for far-field super-resolution imaging. *Nature communications* **3**, 1205. 10.1038/ncomms2176 (2012). 23149749 10.1038/ncomms2176

[5] Li, T. et al. Revolutionary meta-imaging: from superlens to metalens. *Photonics Insights* **2**, R01. 10.3788/PI.2023.R01 (2023).

[6] Synge, E. An application of piezo-electricity to microscopy. *The London, Edinburgh, and Dublin Philosophical Magazine and Journal of Science* **13**, 297–300. 10.1080/14786443209461931 (1932).

[7] Hecht, B. et al. Scanning near-field optical microscopy with aperture probes: Fundamentals and applications. *The Journal of Chemical Physics* **112**, 7761–7774. 10.1063/1.481382 (2000).

[8] Pendry, J. Negative refraction makes a perfect lens. *Physical review letters* **85**, 3966–9. 10.1103/PhysRevLett.85.3966 (2000). 11041972 10.1103/PhysRevLett.85.3966

[9] Fang, N., Lee, H., Sun, C. & Zhang, X. Sub-diffraction-limited optical imaging with a silver superlens. *Science (New York, N.Y.)* **308**, 534–7, 10.1126/science.1108759 (2005). 10.1126/science.1108759 15845849

[10] Durant, S., Liu, Z., Steele, J. & Zhang, X. Theory of the transmission properties of an optical far-field superlens for imaging beyond the diffraction limit. *Journal of the Optical Society of America B* **23**, 2383–2392. 10.1364/JOSAB.23.002383 (2006).

[11] Jacob, Z., Alekseyev, L. V. & Narimanov, E. Optical hyperlens: Far-field imaging beyond the diffraction limit. *Opt. Express* **14**, 8247–8256. 10.1364/OE.14.008247 (2006). 19529199 10.1364/oe.14.008247

[12] Salami, P. & Yousefi, L. Far-field subwavelength imaging using phase gradient metasurfaces. *Journal of Lightwave Technology* **PP**, 1–1, 10.1109/JLT.2019.2902544 (2019).

[13] Akbari-Chelaresi, H., Salami, P. & Yousefi, L. Far-field sub-wavelength imaging using high-order dielectric continuous metasurfaces. *Optics Express* **30**, 10.1364/OE.470221 (2022). 10.1364/OE.470221 36258453

[14] Salami, P. & Yousefi, L. Super-resolution far-field sub-wavelength imaging using multiple holography. *Journal of the Optical Society of America B* **38**, 10.1364/JOSAB.405022 (2021).

[15] Salami, P. & Yousefi, L. Far-field imaging beyond the diffraction limit using waves interference. *Journal of Lightwave Technology* **PP**, 1–1, 10.1109/JLT.2020.2966735 (2020).

[16] Zeng, J., Dong, Y., Wang, Y., Zhang, J. & Wang, J. Optical imaging using orbital angular momentum: Interferometry, holography and microscopy. *J. Lightwave Technol.* **41**, 2025–2040 (2023).

[17] Fürhapter, S., Jesacher, A., Bernet, S. & Ritsch-Marte, M. Spiral phase contrast imaging in microscopy. *Optics Express* **13**, 689–694. 10.1364/OPEX.13.000689 (2005). 19494929 10.1364/opex.13.000689

[18] Ritsch-Marte, M. Orbital angular momentum light in microscopy. *Philosophical Transactions of The Royal Society A Mathematical Physical and Engineering Sciences* **375**, 20150437. 10.1098/rsta.2015.0437 (2017). 10.1098/rsta.2015.0437 PMC5247481 28069768

[19] Kozawa, Y., Matsunaga, D. & Sato, S. Superresolution imaging via superoscillation focusing of a radially polarized beam. *Optica* **5**, 86. 10.1364/OPTICA.5.000086 (2018).

[20] Cheng, K., Li, Z., Wu, J., Hu, Z.-D. & Wang, J. Super-resolution imaging based on radially polarized beam induced superoscillation using an all-dielectric metasurface. *Optics Express* **30**, 10.1364/OE.446481 (2022). 10.1364/OE.446481 35209411

[21] Li, L. & Li, F. Beating the rayleigh limit: Orbital-angular-momentum-based super-resolution diffraction tomography. *Physical review. E, Statistical, nonlinear, and soft matter physics* **88**, 033205, 10.1103/PhysRevE.88.033205 (2013). 10.1103/PhysRevE.88.033205 24125378

[22] Du, X. et al. Full-color quasi-achromatic imaging with a dual-functional metasurface. *Nano letters*10.1021/acs.nanolett.5c00695 (2025). 40248883 10.1021/acs.nanolett.5c00695

[23] Yang, H. et al. Metasurface higher-order poincaré sphere polarization detection clock. *Light, science & applications* **14**, 63. 10.1038/s41377-024-01738-1 (2025). 10.1038/s41377-024-01738-1 PMC11762790 39863612

[24] Yang, H. et al. All-dielectric metasurface for fully resolving arbitrary beams on a higher-order poincaré sphere. *Photonics Research* **9**, 10.1364/PRJ.411503 (2021).

[25] Li, X. et al. Monolithic spiral metalens for ultrahigh-capacity and single-shot sorting of full angular momentum state. *Advanced Functional Materials* **34**, 10.1002/adfm.202311286 (2023).

[26] Guo, Y. et al. Spin-decoupled metasurface for simultaneous detection of spin and orbital angular momenta via momentum transformation. *Light: Science & Applications* **10**, 10.1038/s41377-021-00497-7 (2021). 10.1038/s41377-021-00497-7 PMC7994415 33767137

[27] Zhang, Z. et al. Robust measurement of orbital angular momentum of a partially coherent vortex beam under amplitude and phase perturbations. *Opto-Electronic Science* **3**, 240001–240001, 10.29026/oes.2024.240001 (2024).

[28] Chen, R., Zhou, H., Moretti, M., Wang, X. & Li, J. Orbital angular momentum waves: Generation, detection and emerging applications. *IEEE Communications Surveys & Tutorials* **PP**, 1–1, 10.1109/COMST.2019.2952453 (2019).

[29] Devlin, R. C., Ambrosio, A., Rubin, N. A., Mueller, J. P. B. & Capasso, F. Arbitrary spin-to–orbital angular momentum conversion of light. *Science* **358**, 896–901. 10.1126/science.aao5392 (2017). 29097490 10.1126/science.aao5392

[30] Arbabi, A., Horie, Y., Bagheri, M. & Faraon, A. Dielectric metasurfaces for complete control of phase and polarization with subwavelength spatial resolution and high transmission. *Nature nanotechnology* **10**, 10.1038/nnano.2015.186 (2015). 10.1038/nnano.2015.186 26322944

[31] Piccardo, M. & Ambrosio, A. Arbitrary polarization conversion for pure vortex generation with a single metasurface. *Nanophotonics* **1**, 10.1515/nanoph-2020-0332 (2020).

[32] Abed, O. & Yousefi, L. Tunable metasurfaces using phase change materials and transparent graphene heaters. *Optics Express* **28**, 33876. 10.1364/OE.404103 (2020). 33182867 10.1364/OE.404103

[33] Ra’di, Y., Nefedkin, N., Popovski, P. & Alù, A. Metasurfaces for next-generation wireless communication systems. *National Science Review* **10**, 10.1093/nsr/nwad140 (2023). 10.1093/nsr/nwad140 PMC10306355 37389140

[34] Taravati, S. & Eleftheriades, G. Full-duplex reflective beamsteering metasurface featuring magnetless nonreciprocal amplification. *Nature Communications* **12**, 4414, 10.21203/rs.3.rs-139662/v1 (2021). 10.1038/s41467-021-24749-7 PMC8292412 34285230

[35] Shameli, M. A., Fallah, A. & Yousefi, L. Developing an optimized metasurface for light trapping in thin-film solar cells using a deep neural network and a genetic algorithm. *Journal of the Optical Society of America B* **38**, 10.1364/JOSAB.432989 (2021).

[36] Wang, Y. et al. High-efficiency broadband achromatic metalens for near-ir biological imaging window. *Nature Communications* **12**, 5560. 10.1038/s41467-021-25797-9 (2021). 34548490 10.1038/s41467-021-25797-9 PMC8455568

[37] Ding, F. et al. Versatile polarization generation and manipulation using dielectric metasurfaces. *Laser & Photonics Reviews* **14**, 10.1002/lpor.202000116 (2020).

[38] Ding, F., Chen, Y. & Bozhevolnyi, S. Gap-surface plasmon metasurfaces for linear-polarization conversion, focusing and beam splitting. *Photonics Research* **8**, 10.1364/PRJ.386655 (2020).

